# Sexual and reproductive health in Greenland: evaluation of implementing sexual peer-to-peer education in Greenland (the SexInuk project)

**DOI:** 10.3402/ijch.v74.27941

**Published:** 2015-10-28

**Authors:** Anne-Sophie Homøe, Ane-Kersti Skaarup Knudsen, Sigrid Brisson Nielsen, Anna Garcia-Alix Grynnerup

**Affiliations:** 1 Faculty of Health and Medical Sciences, Copenhagen University, Copenhagen N, Denmark; 2 Faculty of Health, Aarhus University, Aarhus C, Denmark

**Keywords:** sexually transmitted infections, Greenlandic students, volunteer work, sexual behaviour

## Abstract

**Background:**

For decades, the rates of sexually transmitted infections (STIs), such as gonorrhoea, chlamydia and syphilis, have increased in Greenland, especially within the young age groups (15–29 years). From 2006 to 2013, the number of abortions has been consistent with approximately 800–900 abortions per year in Greenland, which is nearly as high as the total number of births during the same period. Previous studies in Greenland have reported that knowledge about sexual health is important, both as prevention and as facilitator to stop the increasing rates of STIs. A peer-to-peer education programme about sexual health requires adaption to cultural values and acceptance among the population and government in order to be sustainable.

**Objective:**

Formative evaluation of a voluntary project (SexInuk), in relation to peer-to-peer education with focus on sexual health. Two workshops were conducted in Nuuk, Greenland, to recruit Greenlandic students.

**Design:**

Qualitative design with focus group interviews (FGIs) to collect qualitative feedback on feasibility and implementation of the project. Supplemented with a brief questionnaire regarding personal information (gender, age, education) and questions about the educational elements in the SexInuk project. Eight Greenlandic students, who had completed one or two workshops, were enrolled.

**Results:**

The FGIs showed an overall consensus regarding the need for improving sexual health education in Greenland. The participants requested more voluntary educators, to secure sustainability. The articulation of taboo topics in the Greenlandic society appeared very important. The participants suggested more awareness by promoting the project.

**Conclusion:**

Cultural values and language directions were important elements in the FGIs. To our knowledge, voluntary work regarding peer-to-peer education and sexual health has not been structurally evaluated in Greenland before. To achieve sustainability, the project needs educators and financial support. Further research is needed to investigate how peer-to-peer education can improve sexual and reproductive health in Greenland.

In recent decades, the rates of sexually transmitted infections (STIs), such as gonorrhoea and chlamydia, have been increasing in Greenland (1,2). In 2003–2006, the rates of both infections were reported high in Greenland compared to other Arctic countries, for example, Alaska (USA) and the Northern territories (Canada) (3). The incidence of syphilis in Greenland has increased, from zero cases in 2010 to 85.3 per 100,000 inhabitants in 2014 (4). The stated STIs are mainly affecting the young age groups (15–29 years) (3,4). From 2006 to 2013, the number of abortions has been consistent with approximately 800–900 abortions per year in Greenland, which is nearly as high as the total number of births during the same period (5–9). The women aged between 20 and 24 years accounted for the highest percent of abortions (6,8). The total abortion rate in Greenland differs markedly from other Nordic countries. In 2012, the total abortion rate in Greenland was 2,000 abortions per 1,000 women (aged 15–49 years). In comparison, the total abortion rate in other Nordic countries (Faroe Island, Norway, Denmark, etc.) was less than 700 abortions per 1,000 women (aged 15–49 years) in the same period, see Fig. 1 (10). To our knowledge, the exact number of unintended pregnancies in Greenland is not available in the literature or in statistical reports. In Greenland, it is solely up to the pregnant woman herself to decide whether an abortion is to be performed (11). However, several cultural concerns may be considered: lack of consistent contraception use, short- and long-term consequences of abortion procedure, the husband's wish not to have a child, and a gap between what the women know about contraception and what the health communities believe they can teach them (12,13). In addition, Greenlandic families have a strong influence on whether or not a woman has an abortion (14,15). Regardless of the well-known problems concerning reproductive health in Greenland, the subject has not been prioritized in the Greenlandic Public Health Program (*Inuuneritta II*) 2013–2019 (16).

**Fig. 1:**
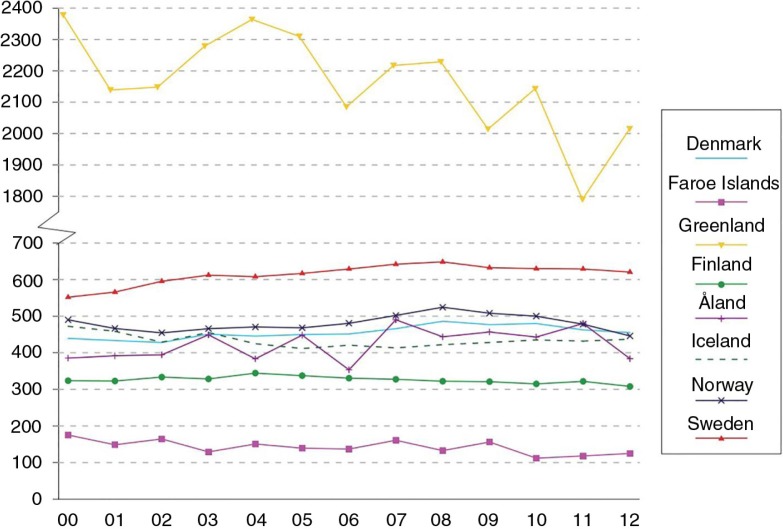
Total abortion rate per 1,000 women (aged 15–49 years) (*y*-axis) per year in 2000–2012 (*x*-axis). Calculated from the age-specific abortion rates for the selected Nordic countries. Reproduced with permission from NOMESCO (10).

Expectedly, awareness of sexual and reproductive health among the youth in Greenland is an important factor to reduce the number of STIs and the abortion rates, respectively (17). Yet, focusing only on providing factual information about sexual health may not be sufficient or effective in reducing negative sexual health outcomes (18). Although sexual health education is mandatory in the public schools in Greenland as a part of the course “Personal Development” (19), no studies show whether all pupils receive adequate and comprehensive sexual health education, with focus on anatomy, contraception, STIs as well as sensitive topics, such as love, sexuality, and relationships. In addition, families in Greenland will postpone or avoid talking about sexual health as it is considered awkward and difficult (20). Still, direct communication regarding these topics can be effective in reducing STIs and promoting sexual health (21). By implementing sexual health education, young people are ensured sufficient knowledge regarding sexual health, and, furthermore, it helps to fight taboos. Sexual health education in the public schools has existed for many years, but within a few years the teaching methods have developed drastically (22,23). Peer-to-peer education, for example, education performed by young people to young people, and a young-individual focused approach is a widespread practice to promote sexual health. Peer-to-peer education is a popular method among young people as it creates a fun and comfortable teaching atmosphere, which allows students to talk actively about sexual health (24). Furthermore, peer-to-peer education introduces visual demonstrations, activity/behaviour games and icebreakers.

Peer-to-peer education regarding sexual health has, to our knowledge, only been tried once before in the town Aasiaat at the Greenlandic west coast. The project was named the Sex Pilots, but whether it was structurally evaluated or implemented in other Greenlandic towns is unknown. In order to create a basic platform for a sexual health educational programme for pupils in the Greenlandic public school system (7–10 grade), the project SexInuk was initiated. The aim was to establish a sustainable project targeted to recruit Greenlandic nursing and teaching students to draw focus to the sexual health issues. These students are relevant to address since they have a general interest in health and education. The method in the SexInuk project is based on peer-to-peer education and was conducted through workshops held by Danish medical students. The idea is to improve the sexual health among young age groups in Greenland by educating Greenlandic students, who then educate the pupils about STIs, anatomy, contraceptives etc. in the native language. Hence, the project is a peer-to-peer education programme. The project is to be implemented at the public schools in Nuuk, Greenland. Since the SexInuk project is voluntary, the commitment from the Greenlandic students is vital for the sustainability of the project in Greenland.

Thus, the objective was to conduct a formative evaluation of the SexInuk project in its pilot phase: primarily, to improve the project in relation to peer-to-peer education with focus on sexual health, and secondly, to adapt the project to cultural values in the future.

## Methods and materials

The SexInuk project was established in 2012. The planning, fundraising and conduction of the workshops was carried out by Danish medical students with experience and knowledge about peer-to-peer education and voluntary work in Denmark. The Danish delegation was responsible for contact and arrangement of cooperative agreements with the Institute of Nursing Science and Health Research in Nuuk – *Peqqissaanermik Ilinniarfik* (PI), the Office of Health & Prevention in Greenland (Early Intervention in Greenland – PAARISA), the Department of Health & Infrastructure at the Greenlandic Home Rule Government and the Greenlandic School principal in Nuuk at Sermersooq municipality.

### Workshops

**Fig. 2:**
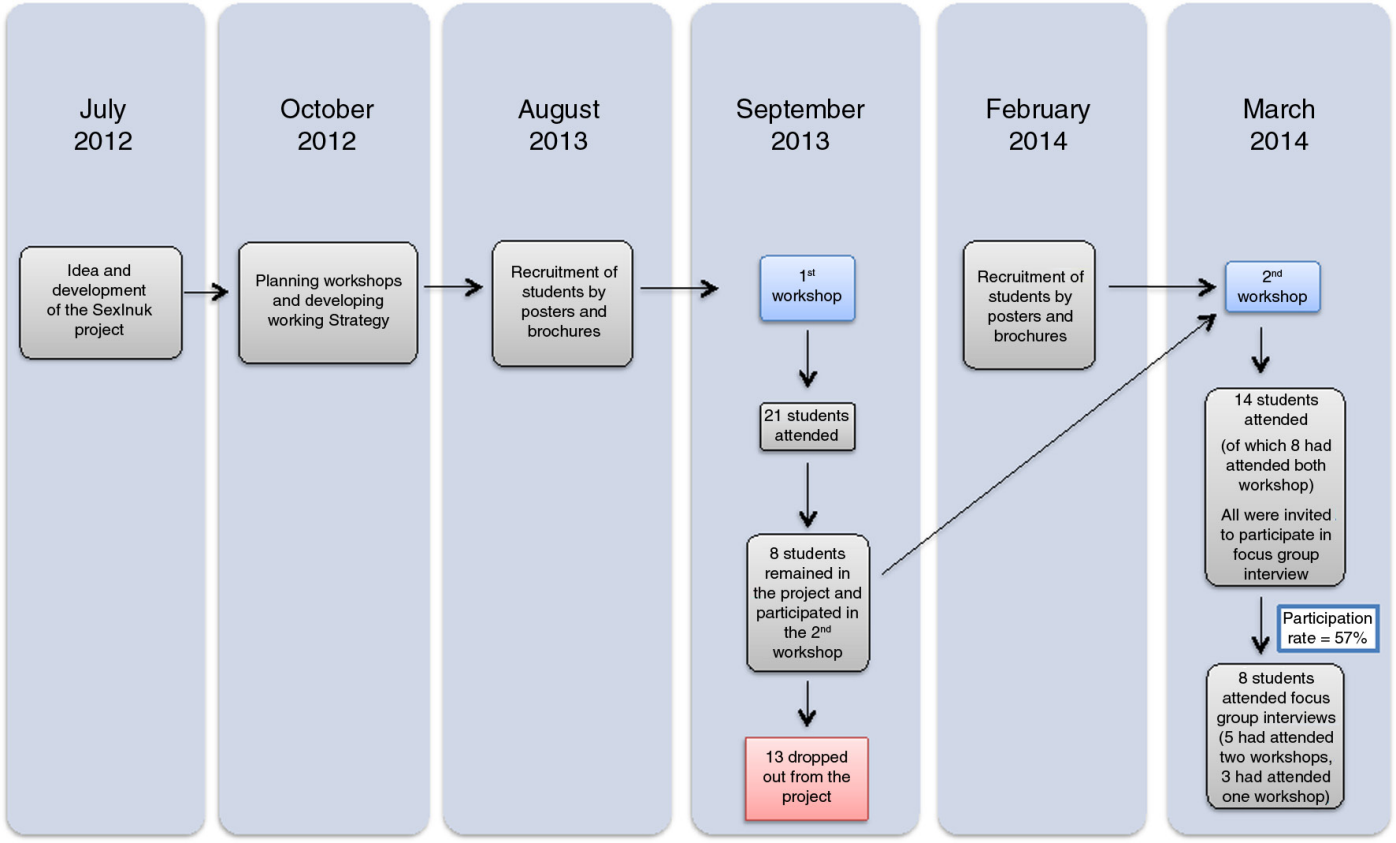
Timeline and flow diagram for inclusion of participants in workshops and focus group interviews.

Two workshops were held at PI, in September 2013 and in March 2014, respectively. The purpose of the workshops was to recruit Greenlandic students to be volunteer peer educators. Prior to the workshops, information was distributed through posters and brochures. Participation of Greenlandic students in the workshops depended on interest in sexual health and awareness of the project via posters and brochures. At the first workshop, the target group was Greenlandic nursing students. Twenty-one Greenlandic students participated in the first workshop (20 nursing students from PI and 1 student from University of Greenland). In the second workshop, the recruitment of students was assigned to the nursing student's responsibility. The participants at the second workshop were primarily nursing students; however, teaching students from the Teacher Seminar in Nuuk and students from the University of Greenland also participated in the second workshop. Hence, the second workshop counted 14 Greenlandic students (8 nursing students, 4 teaching students and 2 students from University of Greenland). Figure 2 shows a timeline med flow diagram for inclusion of participants in workshops and focus group interviews (FGIs). Each of the two workshops lasted 3 days from approximately 1 to 5 pm. The workshops included (a) introduction to the SexInuk project and voluntary work, (b) presentation from a nurse employed at Queen Ingrid's Hospital (abortion in Greenland) and an employee from PAARISA (sexual health problematic in Greenland) and (c) implementation of sexual health education in public schools in Greenland. This way, the Greenlandic students could get an idea of how the main topics (anatomy, contraption and STIs) should be presented by visual demonstrations, and how activity/behaviour games and icebreakers were performed. Icebreakers are simple games or exercises used for introduction during the sexual health education and often have elements of direct learning. The educational methods used were based on a Danish model, recognized by the sexual health education programme in Denmark (25).

### Focus group interview

#### Study population

Fourteen students from the second workshop were invited to an FGI. The aim of the FGI was to conduct a formative evaluation of the SexInuk project, and to collect qualitative feedback on feasibility and implementation of the project. It was not possible to invite the participants from the first workshop, since some participants had dropped out from the project (13 participants), see Fig. 2. Eight female students from the second workshop were recruited to the FGIs, which was our final study population (participation rate 57%). Five of the students had completed both the first and second workshops. All participants had completed 1–9 grade in a Greenlandic public school. The eight students were distributed into two groups depending on which day the students could participate in the FGI, with four people in each of the two groups. The two interview groups are referred to as focus group 1 and focus group 2.

#### Environment

The setting for the FGI was comfortable and familiar, as the classroom used was the same as that used for the second workshop. Participants were instructed to turn off their cell phones, and a note was placed on the door to the room that stated: “Please: Do not disturb – interview in progress.”

#### Moderator and assistant moderator

Two Danish medical students conducted the FGIs. The language throughout the FGI was Danish, since all Greenlandic students spoke and understood Danish. Prior to the FGIs, the moderator explained the role of the moderator and the assistant moderator to the participants. The moderator was responsible for the FGI, while the assistant moderator was responsible for the technical equipment, seating arrangements and taking notes during the FGI. The notes included participants’ body language, distractions in the room, head nodding etc. The moderator used the following approach: welcome, ground rules, overview and topics, presentation of the participants, and then the first questions. To ensure compatibility, predetermined questions (presented in the Questionnaire section and the Result section; Table II) were applied at the FGIs. The purpose was to create a permissive and tolerable atmosphere among the participants in order to ensure a comfortable conversation. The guidelines for the FGI were followed by elementary instructions for designing and conducting FGIs (26,27).

#### Questionnaire

Prior to the FGI, the participants completed a 3-page questionnaire in Danish. It took 5–10 minutes for the participants to complete the questionnaire. The first page contained personal information about age, gender, educational level and participation in one or two workshops. At the second page, a few simple questions were asked, with the participant's subjective experience of greater knowledge regarding anatomy, STIs, contraception and activity/behaviour games or icebreakers, for example, “Did you experience greater knowledge about chlamydia after participating in the SexInuk workshops” (Yes/No/Unchanged/Do not know). Finally, the students had to describe their own experiences with sexual health education in the public school and give any additional comments to the project.

#### Data analysis

The Danish medical students, who conducted the workshops and the FGIs, were responsible for the data analysis. The questionnaire data were analyzed using Excel (Microsoft Office, software edition 2011), due to simple questions and few participants. The FGIs were audio-recorded. Subsequently, the FGI was systematically transcribed using Word (Microsoft Office, software edition 2011) and iTunes (Apple Music, software edition 11.1.5 OS X). Afterwards, the transcripts were read and crosschecked. The transcripts were analyzed by the predetermined questions. The Danish medical students discussed the overall answers. No verbal disagreements occurred among the participants during the FGIs, which allowed the individual answers to be merged. Subsequently, the answers in the two focus groups were compared to find diversities and similar answers. During this procedure, the transcripts were reviewed again.

#### Ethics

The Ethics Committee for medical research in Greenland approved the SexInuk project. Informed consent was signed before enrollment in the FGI. All participants were informed about the purpose of the FGI and accepted participation.

## Results

The demographics of the participants are shown in Table I. The participants in focus group 2 appeared older than the participants in focus group 1. The majority of participants were nursing students (5 out of 8). The four participants in focus group 1 had participated in two workshops, and furthermore, they all had experience with peer-to-peer education in the public school in Greenland, since they had been teaching about sexual health to Greenlandic pupils, which was only the case for one participant in focus group 2.

**Table I:** Demographics of participants in focus groups 1 and 2 (N=8)

Parameters	Focus group 1	Focus group 2
n (gender)	4 (w)	4 (w)
Age*	26.0 (21–31)	33 (31–35)
Education level – n		
Nursing student	3	2
Teaching student	–	2
University degree	1	–
Participating in workshop – n		
In 1 workshop	0	3
In two workshops	4	1
Experience with peer-to-peer education – n		
No	0	3
Yes	4	1

N, total number of participants; n, number of participants in each group; w, women; *, median (min–max); –, no corresponding value.

The brief questionnaire (Table II) regarded the participants’ subjective experiences of greater knowledge regarding certain main topics, for example, anatomy, contraception and STIs, after participation in the workshops. The results show that 7 out of 8 participants stated to have the subjective experience of greater knowledge about STIs. More than half of the participants stated to have the subjective experience of greater knowledge concerning male anatomy and contraceptives. Few of the participants were familiar with activity/behaviour games and icebreakers prior to the workshop. Most of the participants (5 out of 8) had received some kind of sexual health education in public school. However, from their subjective point of view the quality and quantity of the sexual education in the public schools differed considerably.

**Table II:** Questionnaire analysis for participants in focus group interviews (N=8)

Question: Did you experience greater knowledge about (topic) after participating in the SexInuk workshop?				

Topic – n	Yes	No	Unchanged	Don't know
STIs	7	1	0	0
Male anatomy	5	2	1	0
Female anatomy	2	3	2	1
Contraceptives	5	1	2	0
I previously knew about activity/behaviour games	2	5	0	1
I previously knew about icebreakers	2	4	0	2
I have received sexual education in the public school in Greenland	5	2	0	1

N, total number of participants; n, number of answers for the corresponding category; STIs, sexually transmitted infections; icebreaker, simple games or exercises that can be used for introduction during the peer-to-peer education.

A comparison of the two FGIs is provided in Table III along with the predetermined questions. The answers are merged by the participant's individual answers.

**Table III:** Comparison of answers from the focus groups 1 and 2 by merging of individual answers

Predetermined questions	Group	Results	Citations	Comparison
What is a (good) sexual educator?	1	A peer-educator, who can relate to Greenlandic cultural values, and can talk about taboo topics.	“It is important to know how the Greenlandic people behave, so you can educate in the best possible way.”	Knowledge is important, both in terms of the cultural and educational aspects. Importance of articulation of taboo and difficult topics.
	2	A person with experience and knowledge about sexual health. A good facilitator, who can answer difficult questions, and perform actively.	“Sufficient knowledge gives the youth an idea of what sexual health is, since some of them comes from tough homes.”
What is your own personal experience with sexual health education in your schooling in the public school in Greenland?	1	Non-existing at some schools. Poor and uninteresting. Few classes had theme week concerning sexual health.	“My teacher told of menstruation and how women smelled badly because of this… it really hurt some of the girls in our class.”	Missing understanding of how important sexual health education is in the public school. Teachers neglect and have low priority of the subject.
	2	n.a.	n.a.
How was your knowledge concerning anatomy, contraceptive and STIs before participating in the SexInuk workshop(s)?	1	n.a.	n.a.	
	2	Some participants remember few things from their own sexual health education in the public school. Anatomy was refreshed. New knowledge regarding HIV and emergency contraception.	“My knowledge about anatomy has been updated, since I participated in SexInuk… Because I don't remember everything about anatomy as a nurse.”	Most of the participants, had the subjective experience of greater knowledge about several sexual health topics.
What was your thought about SexInuk before participating in the workshop(s)?	1	A necessity to change the attitude and behaviour concerning sexual health. An important prevention project.	“There is ‘rotten’ in Greenland… I have heard horrible stories about sexual health education and how it's performed.”	Both groups agree that changes are needed in Greenland, mainly to improve the sexual and reproductive health and by securing future generations.
	2	Ignorance and pressure among the youth in Greenland.	“Some girls experience pressure concerning losing their virginity at an early age.”
What did work in the organization of the SexInuk workshop(s)	1	External teachers regarding sexual problematic.	“The external teacher from PAARISA who told about sexual abusers, really gave me something to think about.”	The two groups highlight two separate things.
	2	Good to be active and involved during the workshop. Active learning.	“The educational methods were great!”	
What did not work in the organization of the SexInuk workshop(s)?	1	No considerably new knowledge at the second workshop, compared to the first workshop.	“Last time the concept was brand new.”	The two groups highlight two separate things.
	2	The language used in the workshop is very provocative for some people in Greenland.	“The Greenlanders are not used to hearing naughty and provocative words.”	
How would you describe the benefits from the SexInuk workshop(s)?	1	The educational methods and the fundraiser session were good.	“It is important to set a good example.”	Overall consensus concerning more knowledge of educational methods and how to use them in a voluntary context.
	2	The educational methods works.	“The icebreakers are really good.”
How can you use the knowledge about sexual health from the SexInuk workshop(s) in the future?	1	Both privately and professionally.	“I would like to tell about sexual health to the hospital staff, so that they can use it in consultations with patients.”	Focus on sexual health privately and professionally. Important to articulate the subject.
	2	Both privately and professionally.	“I can use it for conversations with friends, my kids or other parents.”	
What is needed to improve the SexInuk project?	1	More volunteer peer educators.	“I'm worried about the project's future, if we don't recruit more voluntary educators.”	More public awareness of the project, so it can be sustainable.
	2	More public relations (PR) to the project. Proliferation in other towns than Nuuk.	“We can have commercials in the Greenlandic TV or in newspapers.”	
On which level in the community do you think learning about sexual health is important?	1	The project needs to be embracing. The government needs to increase the awareness regarding sexual and reproductive subjects.	“We need brave young people, who can take independent decisions… And know when to say NO”. “I am furious at the politicians, they are not paying enough attention to the sexual and reproductive health area.”	The community and government in Greenland need to know more about sexual health to secure the future generations’ reproductive health.
	2	Spread the good message concerning sexual health. Involvement of parents.	“We need more articulation and less taboo.”	

n.a., not available; STIs, sexually transmitted infections.

The two groups agreed that a good sexual educator was a person with knowledge about sexual health and comprehensive understanding of cultural values. Focus group 1 reported from their subjective point of view that sexual health education was non-existing in some public schools. There was great consensus between the two groups regarding thoughts about benefits of the workshops, primarily that behavioural changes are needed in order to improve the sexual health in Greenland. There was a remarkable difference concerning the questions of what methods were useful and what methods were not worthwhile during the workshops. Both focus groups stated that the educational methods were good, and that the participants could use the knowledge from the workshops privately and professionally. The FGIs revealed an overall understanding within the cultural values of sexual health issues and taboo topics in the Greenlandic society. Both groups reflected on the future of the project and gave relevant advice concerning sustainability. According to the Greenlandic students to ensure sustainability, there is a need for more voluntary peer educators and further publicity (posters, TV commercials, etc.) to create more awareness about the project. Also, the participants in FGI 1 discussed the importance of governmental support and the lack of political concern regarding sexual and reproductive health. Furthermore, there was general agreement concerning the need for improving sexual health education in public schools, and overall need for greater knowledge about sexual health in the Greenlandic community.

One of the most encouraging citations during the FGI concerned the youth's future in Greenland:

… where we (peer educators) give them (the Greenlandic youth) a match, so they can light their own candle. We will not light the candle for them, but they can decide to light the candle themselves. Thus, they make their own choice ….

## Discussion

The current study assesses a formative evaluation of the feasibility and implementation regarding a voluntary peer-to-peer education programme about sexual health in Greenland. Sexual and reproductive health in Greenland is an important topic. We believe that the use of peer-to-peer education is a valuable tool when it comes to educating young people about sexual and reproductive health. The educational methods used in the SexInuk project are based on a Danish model (25). However, because of the cultural differences between Greenland and Denmark, caution must be taken when applying the same methods. Thus, it is important to continuously evaluate the project to fit it into Greenlandic settings.

The Greenlandic students experienced a subjective greater knowledge during the workshops, especially about STIs and to some extent male anatomy and contraceptives. Furthermore, the Greenlandic students reported that knowledge about cultural values is important to be a good sexual educator and that taboo topics need to be articulated. The learning methods in the workshops were educational for the Greenlandic students. The participants’ answers provided an insight as to whether the programme is correctly constructed and whether or not it could be adapted to public schools. From our insights, an example of a culturally relevant sexual health education in public schools in Greenland could include the same elements as the Danish model with presentation of anatomy, STIs and contraception; supplemented with simple activity/behaviour games and icebreakers, which the Greenlandic students found educational and fun. Thus, these methods could easily be used in Greenlandic public schools. However, in our experience, it is necessary to respect cultural differences regarding the Greenlandic students’ need for personal space and timidity, but also that the Greenlandic students like to laugh and have fun.

We are now aware of the importance of cultural experiences and understanding when implementing peer-to-peer education in Greenland. The language barrier needs to be respected, as well as concerns regarding recruitment of future peer educators. By using a language that is considered too provocative, you risk, unintentionally, to exceed personal boundaries and create an uncomfortable atmosphere. Our overall recommendation is to organize the education programme to the student/pupil's educational level and to use a combination of formal and/or humourous terms, which can create a natural atmosphere. Thus, we have experienced that simple directions for language use are necessary.

In the last century, cultural values and beliefs about sexuality have changed considerably in Greenland. The small settlements were undisturbed communities with a liberal attitude. Sexuality and intercourse were informal and concerned reproduction, hereby survival of the community. In the recent decades, the Westernization in Greenland has resulted in a dilemma regarding the private love life versus the sexual liberation (28,29). These historical and cultural changes should be considered when teaching sexual health in Greenland. In the SexInuk project, the approach was humble and the Greenlandic students assessed the different activity/behaviour games and icebreakers in the workshops. This allowed us to adjust the project to present cultural values in Greenland.

The main limitations of this study are the recruitment of participants (selective and few) and the data analysis of the qualitative method, which gives certain selection bias and interpretation problems. All participants were women; a gender variation would have been ideal. However, this was not a possibility since only one participant in the second workshop was male. The participants were not asked about ethnicity. Yet, all participants had completed 1–9 grade in the Greenlandic public school, and only one participant had been born outside Greenland. The FGIs were conducted by moderators well known to the participants, because moderators were a part of the Danish delegation. This may have influenced the participants’ answers. Ideally, the moderators should have been unknown to the participants and speaking the native tongue in order to obtain an objective opinion. In addition, the brief questionnaire was vague regarding the simple questions about subjective experience of greater knowledge within anatomy, STIs and contraception. Also, the questionnaire had not been validated or piloted before use, and finally the participants were asked retrospectively. Hence, future work could include a questionnaire with a Likert scale, and a before-and-after evaluation to compare. Due to subjective expressions the qualitative design has its limitations. Some answers in the FGIs were not available, since the participants did not answer the given question, resulting in missing answers for unknown reasons. In addition, a key limitation is the data analysis, since the qualitative data should have been analyzed in a validated analysis programme like Atlas.ti or similar. The workshops were only conducted in Nuuk, which cannot be representative for Greenland as a whole.

We experienced several difficulties regarding the implementation of the project. Meetings and communication are challenged by the geographical distance and time difference between Denmark and Greenland. To achieve sustainability with this project, it is essential to have financial support in order to ensure a strong consistent programme. Sexual and reproductive health needs political awareness, and the participants also emphasized this. One of the main challenges was to maintain consistency from the peer educators. The voluntary involvement from the Greenlandic students decreased when the Danish delegation left Greenland. This low level of commitment can be due to several cofactors such as lack of interest, time management and little experience with voluntary work. Thus, because of lack of experience with volunteer work among the Greenlandic students, sustainability is challenging.

A Greenlandic study called *Inuulluataarneq* (Having the Good Life) implemented a sexual health behavioural intervention in two communities in Greenland (17,30,31). The overall purpose was to reduce STIs. The results reported that a key factor was parent/guardian communication regarding essential sexual health topics. In connection to these findings, our study emphasizes the importance of communication about sensitive sexual health topics in order to decrease taboos and thereby empowering young people to act responsibly regarding sexual health.

The Sex Pilots was a previous sexual programme in Greenland from the mid to late 1990s in the town Aasiaat. It was established by a Danish nurse, PAARISA and the local municipality (32), and was an attempt from the Greenlandic government to provide peer-to-peer led sexual health education. However, to our knowledge the programme has not been structurally evaluated, thus we cannot compare our data concerning this pilot-phase implementation.

An ongoing project named FOXY (Fostering Open eXpression among Youth) (33) has been a great success in the Northwestern Territories in Canada at Nunavut and Yukon. Briefly, the purpose is open dialogue with young women about sexual health, sexuality and relationships:

FOXY a participatory action research project, which means youth are involved with all aspects of the project, from its development to its implementation and the evaluation. The research component of FOXY involves looking at the effectiveness of FOXY for empowering and facilitating dialogue about sexual health issues. (34)

There is a great similarity between the project purpose of FOXY and SexInuk. Still, the major difference is the effort needed by volunteers in SexInuk.

Improved and comprehensive sexual health education is recommended in other Arctic countries (35), to facilitate a positive view on sexual health topics.

In summary, to our knowledge peer-to-peer education has not been structurally evaluated in Greenland before. It is important to emphasize the importance of sexual health education among the Greenlandic youth, in order to reduce the historical high rates of STIs as well as the number of abortions. Knowledge and communication are in our opinion important tools. Furthermore, major sexual health disparities are present, when comparing Greenland with other Nordic countries. This project has recognized that it is possible to raise awareness and to create a well-functioning team of peer educators by recruiting Greenlandic students, who can speak Greenlandic and have knowledge of Greenlandic cultural values. The educational methods used in the project appeared to create an atmosphere of trust and to break down barriers regarding taboo topics. To ensure the success of a sexual health education programme, sustainability, publicity, and general acknowledgement is needed. Thus, there is a need for continuous evaluation of the benefits and limitations of this method in the SexInuk project. Further research is needed to investigate how sexual peer-to-peer education can improve sexual and reproductive health in Greenland.
